# Prospective Observational Study on acute Appendicitis Worldwide (POSAW)

**DOI:** 10.1186/s13017-018-0179-0

**Published:** 2018-04-16

**Authors:** Massimo Sartelli, Gian L. Baiocchi, Salomone Di Saverio, Francesco Ferrara, Francesco M. Labricciosa, Luca Ansaloni, Federico Coccolini, Deepak Vijayan, Ashraf Abbas, Hariscine K. Abongwa, John Agboola, Adamu Ahmed, Lali Akhmeteli, Nezih Akkapulu, Seckin Akkucuk, Fatih Altintoprak, Aurelia L. Andreiev, Dimitrios Anyfantakis, Boiko Atanasov, Miklosh Bala, Dimitrios Balalis, Oussama Baraket, Giovanni Bellanova, Marcelo Beltran, Renato Bessa Melo, Roberto Bini, Konstantinos Bouliaris, Daniele Brunelli, Adrian Castillo, Marco Catani, Asri Che Jusoh, Alain Chichom-Mefire, Gianfranco Cocorullo, Raul Coimbra, Elif Colak, Silvia Costa, Koray Das, Samir Delibegovic, Zaza Demetrashvili, Isidoro Di Carlo, Nadezda Kiseleva, Tamer El Zalabany, Mario Faro, Margarida Ferreira, Gustavo P. Fraga, Mahir Gachabayov, Wagih M. Ghnnam, Teresa Giménez Maurel, Georgios Gkiokas, Carlos A. Gomes, Ewen Griffiths, Ali Guner, Sanjay Gupta, Andreas Hecker, Elcio S. Hirano, Adrien Hodonou, Martin Hutan, Orestis Ioannidis, Arda Isik, Georgy Ivakhov, Sumita Jain, Mantas Jokubauskas, Aleksandar Karamarkovic, Saila Kauhanen, Robin Kaushik, Alfie Kavalakat, Jakub Kenig, Vladimir Khokha, Desmond Khor, Dennis Kim, Jae I. Kim, Victor Kong, Konstantinos Lasithiotakis, Pedro Leão, Miguel Leon, Andrey Litvin, Varut Lohsiriwat, Eudaldo López-Tomassetti Fernandez, Eftychios Lostoridis, James Maciel, Piotr Major, Ana Dimova, Dimitrios Manatakis, Athanasio Marinis, Aleix Martinez-Perez, Sanjay Marwah, Michael McFarlane, Cristian Mesina, Michał Pędziwiatr, Nickos Michalopoulos, Evangelos Misiakos, Ali Mohamedahmed, Radu Moldovanu, Giulia Montori, Raghuveer Mysore Narayana, Ionut Negoi, Ioannis Nikolopoulos, Giuseppe Novelli, Viktors Novikovs, Iyiade Olaoye, Abdelkarim Omari, Carlos A. Ordoñez, Mouaqit Ouadii, Zeynep Ozkan, Ajay Pal, Gian M. Palini, Lars I. Partecke, Francesco Pata, Michał Pędziwiatr, Gerson A. Pereira Júnior, Tadeja Pintar, Magdalena Pisarska, Cesar F. Ploneda-Valencia, Konstantinos Pouggouras, Vinod Prabhu, Padmakumar Ramakrishnapillai, Jean-Marc Regimbeau, Marianne Reitz, Daniel Rios-Cruz, Sten Saar, Boris Sakakushev, Charalampos Seretis, Alexander Sazhin, Vishal Shelat, Matej Skrovina, Dmitry Smirnov, Charalampos Spyropoulos, Marcin Strzałka, Peep Talving, Ricardo A. Teixeira Gonsaga, George Theobald, Gia Tomadze, Myftar Torba, Cristian Tranà, Jan Ulrych, Mustafa Y. Uzunoğlu, Alin Vasilescu, Savino Occhionorelli, Aurélien Venara, Andras Vereczkei, Nereo Vettoretto, Nutu Vlad, Maciej Walędziak, Tonguç U. Yilmaz, Kuo-Ching Yuan, Cui Yunfeng, Justas Zilinskas, Gérard Grelpois, Fausto Catena

**Affiliations:** 1 Department of Surgery, Macerata Hospital, Macerata, Italy; 2 Department of Clinical and Experimental Sciences, University of Brescia, Brescia, Italy; 3 Emergency Surgery, Maggiore Hospital, Bologna, Italy; 4 General Surgery and Polytrauma, San Carlo Borromeo Hospital, Milan, Italy; 5 Department of Biomedical Sciences and Public Health, Unit of Hygiene, Preventive Medicine and Public Health, UNIVPM, Ancona, Italy; 6 Department of Surgery, Bufalini Hospital, Cesena, Italy; 7 General Surgery, University Hospitals Birmingham NHS Foundation Trust, Birmingham, UK; 8 Emergency Surgery, Mansoura University Hospital, Mansoura, Egypt; 9 Emergency Surgery Unit, Maggiore University Hospital, Parma, Italy; 10 Surgery, Kwara State General Hospital, Ilorin, Kwara Nigeria; 11 Surgery, Ahmadu Bello University Teaching Hospital Zaria, Zaria, Nigeria; 12 Surgery, First University Clinic, Tbilisi, Georgia; 13 General Surgery, Baskent Universitesi Adana Eğitim ve Uygulama Hastanesi, Adana, Turkey; 14 General Surgery, Training and Research Hospital of Mustafa Kemal University, Hatay, Turkey; 15 General Surgery, Sakarya University School of Medicine, Sakarya, Turkey; 16 Primary Care, Primary Health Care Centre of Kissamos, Chania, Greece; 17 Department of General Surgery, Medical University of Plovdiv, UMHAT Eurohospital, Plovdiv, Bulgaria; 18 General Surgery, Hadassah Medical Center, Jerusalem, Israel; 19 Surgical Department, Agios Savvas Anticaner Hospital, Athens, Greece; 20 General Surgery, Hospital Habib Bouguefa de Bizerte, Bizerte, Tunisia; 21 General Surgery, S.S. Annunziata Hospital, Taranto, Italy; 22 Surgery, Hospital San Juan de Dios de La Serena, La Serena, Chile; 23 General Surgery, Centro Hospitalar São João, Porto, Portugal; 24 General and Emergency Surgery, San Giovanni Bosco Hospital, Turin, Italy; 25 Surgical Department, General Hospital of Larissa, Larissa, Greece; 26 Chirurgia, Città di Castello Hospital, Città di Castello, Italy; 27 Department of Surgery, Harbor-UCLA Medical Center, Torrance, USA; 28 DEA, La Sapienza Università di Roma, Policlinico Umberto I, Rome, Italy; 29 General Surgery, Kuala Krai Hospital, Kuala Krai, Kelantan Malaysia; 30 Surgery and Obstetrics/Gynaecology, Regional Hospital, Limbe, Cameroon; 31 Emergency Surgery, Azienda Ospedaliera Universitaria Policlinico Paolo Giaccone, Palermo, Italy; 32 Trauma/Acute Care Surgery, University of California San Diego, San Diego, USA; 33 General Surgery, Samsun Training and Research Hospital, Samsun, Turkey; 34 Surgery, CHVNG/E, EPE, Vila Nova de Gaia, Portugal; 35 General Surgery, Numune Training and Research Hospital, Adana, Turkey; 36 Colorectal Surgery, Clinic for Surgery, University Clinical Center Tuzla, Tuzla, Bosnia and Herzegovina; 37 Surgery, Kipshidze Central University Hospital, Tbilisi, Georgia; 38 Surgery, Hamad General Hospital, Doha, Qatar; 39 General and Emergency Surgery, Riga East University Hospital “Gailezers”, Riga, Latvia; 40 General Surgery, Bahrain Defence Force Hospital, Manama, Bahrain; 41 Department of General Surgery, Trauma and Emergency Surgery Division, ABC Medical School, Santo Andreì, SP Brazil; 42 General Surgery, Hospital Garcia de Orta, Lisbon, Portugal; 43 Division of Trauma Surgery, Hospital de Clinicas, University of Campinas (Unicamp), Campinas, Brazil; 44 Department of Abdominal Surgery, Vladimir City Clinical Hospital of Emergency Medicine, Vladimir, Russia; 45 General Surgery Department, Mansoura Faculty of Medicine, Mansoura University, Mansoura, Egypt; 46 Cirugía General y del Aparato Digestivo, Hospital Universitario Miguel Servet, Zaragoza, Spain; 47 Second Department of Surgery, Aretaieion University Hospital, National and Kapodistrian University of Athens, Athens, Greece; 48 Surgery, Therezinha de Jesus University Hospital, Juiz de Fora, Brazil; 49 Upper GI/General Surgery, University Hospitals Birmingham NHS Foundation Trust, Birmingham, UK; 50 Department of General Surgery, Karadeniz Technical University, Farabi Hospital, Trabzon, Turkey; 51 Surgery, Government Medical College and Hospital, Chandigarh, India; 52 Department of General and Thoracic Surgery, University Hospital, Giessen, Germany; 53 Chirurgie, Chud-Borgou/Alibori, Parakou, Benin; 54 Surgical Department, Landesklinikum Hainburg, Hainburg An Der Donau, Austria; 55 4th Surgical Department, Aristotle University of Thessaloniki, Thessaloniki, Greece; 56 General Hospital “George Papanikoalou”, Thessaloniki, Greece; 57 General Surgery, Erzincan University Mengucek Gazi Training and Research Hospital, Erzincan, Turkey; 58 General Surgery, City Hospital #1, Moscow, Russia; 59 Surgery, S M S Medical college, Jaipur, India; 60 Department of Surgery, Hospital of Lithuanian University of Health Sciences Kaunas Clinics, Kaunas, Lithuania; 61 Clinic for Emergency Surgery, Faculty of Medicine University of Belgrade, Belgrade, Serbia; 62 Division of Digestive Surgery and Urology, Turku University Hospital, Turku, Finland; 63 General Surgery, Jubilee Mission Medical College & Research Institute, Thrissur, India; 64 3rd Department of General Surgery, Jagiellonian University Medical College, Krakow, Poland; 65 Emergency surgery, City Hospital, Mozyr, Belarus; 66 Acute Care Surgery, LAC+USC Medical Center, California, USA; 67 Department of Surgery, Inje University Ilsan Paik Hospital, Goyang, Republic of Korea; 68 Department of Surgery, Edendale Hospital, Pietermaritzburg, South Africa; 69 General Surgery, Scarborough, York Teaching Hospital, Scarborough, UK; 70 General Surgery, Colorectal Unit, Hospital de Braga, Braga, Portugal; 71 General and Digestive Surgery, Hospital Fundación Jimenez Diaz, Madrid, Spain; 72 Surgical Disciplines, Regional Clinical Hospital, Kaliningrad, Russia; 73 Faculty of Medicine, Department of Surgery, Siriraj Hospital, Mahidol University, Bangkok, Thailand; 74 Department of Gastrointestinal Surgery, Insular University Hospital of Gran Canaria, Las Palmas, Spain; 75 1st Department of Surgery, Kavala General Hospital, Kavala, Greece; 76 2nd Department of Surgery, Jagiellonian University Medical College, Krakow, Poland; 77 Clinic of surgery, Department of Gastrointestinal Surgery, University Hospital Centre Zagreb, Zagreb, Croatia; 78 Surgical Department, Konstantopouleio General Hospital, Athens, Greece; 79 First Department of Surgery, Tzaneio, Piraeus, Greece; 80 Department of General and Digestive Surgery, Hospital Universitario Doctor Peset, Valencia, Spain; 81 Department of General Surgery, Post-Graduate Institute of Medical Sciences, Rohtak, India; 82 Department of Surgery, Radiology, Anaesthetics and Intensive Care, University of the West Indies, Kingston, Jamaica; 83 University Hospital of the West Indies, Kingston, Jamaica; 84 Department of Surgery Second Surgical Clinic, Emergency Hospital of Craiova, Craiova, Romania; 85 Department of General Surgery and Emergency Medicine, University Hospital, Kraków, Poland; 86 3rd Department of Surgery, Ahepa University Hospital, Thessaloniki, Greece; 87 3rd Department of Surgery, Attikon University Hospital, National and Kapodistrian University of Athens, Medical School, Athens, Greece; 88 General Surgery, Khartoum Teaching Hospital, Khartoum, Sudan; 89 Department of Visceral, Digestive and Oncologic Surgery, Clinique Sainte Marie, Cambrai, France; 90 General Surgery, K R Hospital, Mysore, India; 91 General Surgery, Emergency Hospital of Bucharest, Bucharest, Romania; 92 General Surgery, Lewisham and Greenwich NHS Trust, London, UK; 93 General, Emergency Surgery, Infermi Hospital, Rimini, Italy; 94 Surgery, University of Ilorin Teaching Hospital, Ilorin, Nigeria; 95 General Surgery, King Abdullah University Hospital, Irbid, Jordan; 96 Division of Trauma and Acute Care Surgery, Department of Surgery, Fundación Valle del Lili and Universidad del Valle, Cali, Colombia; 97 Surgery Departement, Medical School of Fezm, Sidi Mohamed Benabdellah University, Fez, Morocco; 98 General Surgery, Elazig Training and Research Hospital, Elazig, Turkey; 99 General Surgery, King George’s Medical University, Lucknow, India; 100 Surgery, University Hospital, Greifswald, Germany; 101 Department of General Surgery, Sant’Antonio Abate Hospital, Gallarate, Italy; 102 Department of Emergency Surgery and Trauma Centre, University Hospital, Kraków, Poland; 103 Surgery and Anatomy, Clinics Hospital, Ribeirão Preto, Brazil; 104 Abdominal surgery, UMC Ljubljana, Ljubljana, Slovenia; 105 Department of Endoscopic, Metabolic and Soft Tissue Tumors Surgery, The University Hospital in Krakow, Kraków, Poland; 106 General Surgery, Hospital Civil de Guadalajara “Dr. Juan I. Menchaca”, Guadalajara, Mexico; 107 Surgery, Bharati Vidyapeeth Deemed University Medical College & Hospital, Sangli, Maharashtra India; 108 Laparoscopic and Metabolic Surgery, KIMS Hospital, Cochin, India; 109 Digestive Surgery, CHU Amiens-Picardie, Amiens, France; 110 General Surgery, Hospital Municipal Dr. Jose de Carvalho Florence, Sao Jose Dos Campos, Brazil; 111 General Surgery, Hospital General Regional # 1 I.M.S.S, Cuernavaca, Mexico; 112 Acute Care Surgery, North Estonia Medical Center, Tallinn, Estonia; 113 General Surgery, University Hospital St George, Plovdiv, Bulgaria; 114 General Surgery, Russells Hall Hospital, Birmingham, UK; 115 General Surgery, Tan Tock Seng Hospital, Singapore, Singapore; 116 Surgery, Hospital & Oncological Centre Novy Jicin, Novy Jicin, Czech Republic; 117 General Surgery, Clinical Hospital at Chelyabinsk Station OJSC “Russian Railways”, Chelyabinsk, Russian Federation; 118 3rd Department of Surgery, IASO General Hospital, Athens, Greece; 119 General Surgery and Polytrauma, University Hospital, Medical College, Jagiellonian University, Kraków, Poland; 120 Cirurgia do Trauma, Hospital Escola Padre Albino, Catanduva, Brazil; 121 Surgery Department #2, Tbilisi State Medical University, Tbilisi, Georgia; 122 General Surgery, Trauma University Hospital, Tirana, Albania; 123 1st Department of Surgery—Department of Abdominal Thoracic Surgery and Traumatology, General University Hospital, Prague, Czech Republic; 124 First Surgical Clinic, St. Spiridon University Hospital, Iasi, Romania; 125 Emergency Surgery, S. Anna Teaching Hospital, Ferrara, Italy; 126 Digestive and Endocrinal Surgery, University Hospital, Angers, France; 127 Department of Surgery, Medical School University of Pécs, Pécs, Hungary; 128 Surgery, ASTS Spedali Civili Brescia, Montichiari, Italy; 129 Department of General, Oncological, Metabolic and Thoracic Surgery, Military Institute of Medicine in Warsaw, Warsaw, Poland; 130 Department of General Surgery, Kocaeli University, Kocaeli, Turkey; 131 Trauma and Emergency Surgery, Chang Gung Memorial Hospital, Taoyuan, Taiwan; 132 Department of Surgery, Tianjin Nankai Hospital, Tianjin, China

**Keywords:** Acute appendicitis, Diagnosis, Management, Surgery, Antibiotics

## Abstract

**Background:**

Acute appendicitis (AA) is the most common surgical disease, and appendectomy is the treatment of choice in the majority of cases. A correct diagnosis is key for decreasing the negative appendectomy rate. The management can become difficult in case of complicated appendicitis. The aim of this study is to describe the worldwide clinical and diagnostic work-up and management of AA in surgical departments.

**Methods:**

This prospective multicenter observational study was performed in 116 worldwide surgical departments from 44 countries over a 6-month period (April 1, 2016–September 30, 2016). All consecutive patients admitted to surgical departments with a clinical diagnosis of AA were included in the study.

**Results:**

A total of 4282 patients were enrolled in the POSAW study, 1928 (45%) women and 2354 (55%) men, with a median age of 29 years. Nine hundred and seven (21.2%) patients underwent an abdominal CT scan, 1856 (43.3%) patients an US, and 285 (6.7%) patients both CT scan and US. A total of 4097 (95.7%) patients underwent surgery; 1809 (42.2%) underwent open appendectomy and 2215 (51.7%) had laparoscopic appendectomy. One hundred eighty-five (4.3%) patients were managed conservatively. Major complications occurred in 199 patients (4.6%). The overall mortality rate was 0.28%.

**Conclusions:**

The results of the present study confirm the clinical value of imaging techniques and prognostic scores. Appendectomy remains the most effective treatment of acute appendicitis. Mortality rate is low.

## Background

Acute appendicitis (AA) is the most common surgical disease with a lifetime risk of 7–8% [1]. Traditionally, appendectomy has been the treatment of choice for acute appendicitis. Mortality rate after appendectomy is very low and may range from 0.07 to 0.7% rising to 0.5 to 2.4% in patients without and with perforation [2, 3]. Furthermore, overall postoperative complication rates ranged between 10 and 19% for uncomplicated AA and reaching 30% in cases of complicated AA.

Improving the diagnostic pathway is the cornerstone for decreasing the negative appendectomy rate and the risk of wrong diagnosis. Before the wide spread use of CT scans, the diagnosis of acute appendicitis was mainly based on symptoms, signs, and laboratory data.

Several diagnostic scoring systems for acute appendicitis have been described. The most commonly used are the Alvarado score and AIR—Appendicitis Inflammatory Response (Andersson) score [4, 5]. Both of these scoring systems can increase the diagnostic accuracy, thus guiding the decision-making and decreasing the need of potentially harmful and expensive imaging. In view of the potentially higher morbidity associated with open appendectomy, several authors have proposed less invasive management. Although many controversies exist regarding non-operative management of AA, antibiotics play an important role in the treatment of patients with AA as demonstrated by several prospective trials and meta-analyses [6–14]. AA successfully treated with antibiotics remains a potential cause of recurrent appendicitis. Postoperative wound infections and post-appendectomy adhesional bowel obstruction occurring many decades after the index surgery are commonly described sequalae of appendectomies. Therefore, the comparison of surgery and antibiotic therapy still represent a challenging and debated issue.

The effort to distinguish non-complicated from complicated appendicitis is paramount in ensuring appropriate patient management. Utilizing a CT scan to diagnose cases of suspected AA has been demonstrated, it has high sensitivity (0.99) and specificity (0.95) [15–17]. However, even a CT scan struggles to differentiate between uncomplicated and complicated appendicitis [18, 19].

In the last decade, the laparoscopic approach has overtaken open surgery for most surgeons worldwide in the treatment of both simple and complicated AA. However, it is not yet unanimously considered the “gold standard” in the management of AA because of its higher operative time, increased intra-abdominal abscess risk, and higher costs compared to open appendectomy. Several meta-analyses of prospective randomized trials were performed in an attempt to define the role of laparoscopic appendectomy [20–25]. Literature reports that 2 to 7% of appendicitis tend to present with complex features such as a phlegmon or peri-appendicular abscess [26, 27]. Various published papers suggest treating such patients conservatively, by such methods as ultrasound-guided percutaneous drainage and antibiotic therapy, followed by delayed interval appendectomy [28–31]. The role of appendectomy after successful drainage and resolution of clinical symptoms is even more controversial than percutaneous drainage. The recommendation for interval appendectomy is based on the risk of recurrence and risk of missing an underlying malignancy [32]. However, the recurrence rate has been reported by several studies to be around 7%, reassuringly low; thus, according to some authors, after successful conservative treatment, an interval appendectomy may not be always necessary [32–37].

Recently, a new AA grading system has been proposed by the World Society of Emergency Surgery (WSES). This new grading system is based on clinical presentation, imaging, and surgical findings and aims to provide a standardized classification system based on a uniform patient stratification. The new scoring system intends to aid in determining the optimal post-appendectomy management according to the grade of severity and ultimately contribute to clinical research in appendicitis [38].

Herein we report the results of a prospective observational multicenter worldwide study on AA conducted on behalf of the WSES. To the best of our knowledge, this is the first large-scale observational study on AA performed in institutions from different countries.

## Methods

### Aim

The primary aim of the POSAW study is to describe the clinical, diagnostic, treatment, and pathological profile of patients with AA in surgical departments of worldwide hospitals.

### Study design

This prospective multicenter observational study was performed in 116 worldwide surgical departments from 44 countries over a 6-month period (April 1, 2016—September 30, 2016). All consecutive patients admitted to surgical departments with clinical diagnosis of AA were included in the study. Patient demographics included the following: age, sex, previous episodes of suspected appendicitis, comorbidities (immunosuppression, severe cardiovascular disease, Charlson Comorbidity Index (CCI)) [39], previous antimicrobial therapy, clinical data (axillary temperature, diffuse tenderness, right lower quadrant pain, right lower quadrant tenderness, vomiting) and laboratory findings at admission (white blood count (WBC) and C-reactive protein (CRP)), radiological diagnosis (ultrasound (US) and computer tomography (CT) findings), Alvarado Score, Andersson’s Score [4, 5], type of surgical treatment and adequate source control, WSES Grading System [38], type and duration of antimicrobial therapy, collection of peritoneal swab, microorganisms isolated, admission to intensive care unit (ICU), duration of hospitalization, re-operation, management of postoperative complications at days 7 and 30, Clavien-Dindo Score [40], histopathological findings, and mortality. All patients were monitored until they were discharged or transferred to another ward. The center coordinator of each participating medical institution collected and compiled clinical data in an online case report database. Differences in local surgical practice of each center were respected, and no changes were impinged on local management strategies. Each center followed its own ethical standards and local rules. The study was monitored by a coordinating center, which processed and verified any missing or unclear data submitted to the central database. The study did not attempt to change or modify the clinical practice of the participating physicians: neither informed consent nor formal approval by a local ethics committee was required because of the purely observational nature of the study. The data was completely anonymized, and no patient or hospital information was collected in the website. The study protocol was approved by the board of the WSES, and the study was conducted under its supervision. The board of the WSES grants the proper ethical conduct of the study.

### Inclusion criteria

All patients with suspected clinical diagnosis of AA confirmed by imaging and seen by a surgeon were included in the study.

### Statistical analysis

Data were analyzed in absolute frequency and percentage, in the case of qualitative variables. Quantitative variables were analyzed as medians and interquartile range (IQR). Univariate analyses were performed to study the association between risk factors and in-hospital mortality using a chi-square test, or a Fisher’s exact test, if the expected value of a cell was < 5. All tests were two-sided, and *p* values of 0.05 were considered statistically significant. To investigate factors associated with death, we constructed a logistic regression model, including variables with *p* < 0.05 in the univariate analysis. All statistical analyses were performed using Stata 11 software package (StataCorp, College Station, TX, USA).

## Results

### Patients and diagnosis

A total of 4282 patients were enrolled in the POSAW study. They included 1928 (45%) women and 2354 (55%) men, with a median age of 29 years (IQR, 21–44). 427 (10%) patients had previous episodes of AA. Seventy-one (1.7%) patients were immunosuppressed, and 154 (3.6%) patients suffered from severe cardiovascular disease. Three thousand six hundred seventy (85.7%) patients had no comorbidities (CCI = 0), 589 (13.8%) patients had a CCI between 1 and 5, and in 23 (0.5%), the CCI was greater than 5. 327 (7.6%) where patients received an antimicrobial therapy in the previous 30 days. Clinical and laboratory findings are reported in Table 1.

**Table 1:** Clinical and laboratory findings

Clinical and laboratory findings	Patients *N* = 4282 (100%)
Diffuse tenderness	502 (11.7%)
Right lower quadrant pain	3906 (91.2%)
Right lower quadrant tenderness	2980 (69.6%)
Vomiting	1798 (42.0%)
Temperature > 38 °C	1057 (24.7%)
WBC > 10,000/ml	3494 (81.6%)
CRP < 10 mg/l	848 (19.8%)
CRP 10–50 mg/l	1097 (25.6%)
CRP > 50 mg/l	876 (20.5%)
CRP not reported	1461 (34.1%)

*WBC* white blood count, *CRP* C-reactive protein

Nine hundred and seven (21.2%) patients underwent abdominal CT scan, 1856 (43.3%) patients had an US, 285 (6.7%) patients both CT scan and US, and 1234 (28.8%) patient did not undergo any radiological investigation during hospitalization. Three thousand eight hundred fifty-seven (90.1%) patients had their Alvarado Score recorded, with a median value of 7 (IQR, 6–8). The Alvarado score was ≤ 4 in 460 (11.9%) patients, between 5 and 6 in 1067 (27.7%) patients, between 7 and 8 in 1614 (41.8%) patients, and between 9 and 10 in 716 (18.6%) patients. Three thousand seven hundred fifty-one (87.6%) patients had their Andersson’s Score recorded, with a median value of 6 (IQR, 5–8). In 709 (18.9%) patients, the Andersson’s score was ≤ 4, between 5 and 8 in 2423 (64.6%) patients, and between 9 and 12 in 619 (16.5%) patients. The Alvarado Score was ≥ 5 in 3132 (89.8%) cases of AA confirmed by pathologic exam (RR = 1.11, 1.07–1.15 CI 95%, *p* < 0.001), while Andersson’s Score was ≥ 5 in 2736 (83.4%) cases of AA confirmed by histopathology (RR = 1.11, 1.07–1.14 CI 95%, *p* < 0.001).

### Management

In 3764 (87.9%) patients, WSES Grading System was recorded. One hundred forty-five (3.8%) patients had grade 0, while 1896 (50.4%) had grade 1, 632 (16.8%) grade 2a, 129 (3.4%) grade 2b, 332 (8.8%) grade 3a, 181 (4.8%) grade 3b, 73 (1.9%) grade 3c, and 376 (10.0%) grade 4.

A total of 4097 (95.7%) patients underwent surgery, of which 1809 (42.2%) underwent open appendectomy and 2215 (51.7%) laparoscopic appendectomy, 19 (0.5%) had open lavage and drainage, 4 (0.1%) had laparoscopic lavage and drainage, 29 (0.7%) had an open ileocaecal resection, 3 (0.1%) had laparoscopic ileocaecal resection, 10 (0.2%) underwent percutaneous drainage, and 8 (0.2%) had other surgical procedures. One hundred eighty-five (4.3%) patients did not undergo any surgical intervention, 48 of these patients had uncomplicated appendicitis.

A total of 3463 (80.9%) patients received antibiotics during the hospitalization, which was monotherapy in the case of 1335 (38.6%) patients (Table 2). The median duration of the antimicrobial therapy was 4 days (IQR, 2–7). Among the 3463 patients who received antibiotics, 583 patients received them as antibiotic prophylaxis.

**Table 2:** Antimicrobial therapy administered during hospitalization in 3463 patients

Patients receiving antibiotics	*N* = 3463 (%)
Metronidazole or ornidazole	2021 (58.2%)
Third-generation cephalosporins	1280 (37.0%)
Second-generation cephalosporins	598 (17.2%)
Penicillins + beta lactams-inhibitors	500 (14.4%)
First-generation cephalosporins	437 (12.6%)
Second-generation quinolones	304 (8.8%)
Aminoglycosides	289 (8.3%)
Ureidopenicillins + beta lactams-inhibitors	130 (3.7%)
Aminopenicillins	61 (1.8%)
Carbapenems	58 (1.7%)
Third-generation cephalosporins + beta lactams-inhibitors	52 (1.5%)
Third-generation quinolones	18 (0.5%)
Fourth-generation cephalosporins	12 (0.3%)
Others	15 (0.4%)

*n* number of patients receiving antibiotic treatment

Intraperitoneal microbiological swab was collected from 803 patients (803/4097, 19.6%) who underwent surgical intervention, resulting in 275 (34.2%) positive cultures. The aerobic and anaerobic bacteria identified in samples of peritoneal fluid are reported in Table 3. Additionally, four *Candida albicans* isolates were also identified.

**Table 3:** Aerobic and anaerobic bacteria identified in 275 swabs of peritoneal fluid

Total	*N* = 275
Aerobic gram-negative bacteria	
*Escherichia coli*	159 (57.8%)
*Klebsiella pneumoniae*	19 (6.9%)
*Klebsiella oxytoca*	4 (1.5%)
*Enterobacter* spp.	5 (1.8%)
*Proteus* spp.	1 (0.4%)
*Pseudomonas aeruginosa*	5 (1.8%)
Aerobic gram-positive bacteria	
*Enterococcus faecalis*	42 (15.3%)
*Streptococci* spp.	24 (8.7%)
*Enterococcus faecium*	11 (4.0%)
*Staphylococcus aureus*	6 (2.2%)
Others	7 (2.5%)
Anaerobic bacteria	
*Bacteroides* spp.	105 (38.2%)
*Clostridium* spp.	4 (1.5%)

*n* number of specimens collected

### Outcome

The median length of hospital stay was 3 days (IQR, 2–5). One hundred and seventy-two patients (4%) had a 1-day hospital stay.

In the early postoperative phase, 184 (184/4097, 2.5%) patients were admitted to the ICU. Fifty-four (54/4097, 1.3%) patients underwent re-laparotomy. A total of 3631 (3631/4097, 88.6%) appendixes were analyzed by histopathology, with the following reports: 144 (4%) were normal appendixes, 236 (6.5%) showed evidence of periappendicitis, 1147 (31.6%) of inflammation, 1176 (32.4%) were suppurative appendixes, 255 (7.0%) showed evidence of perforation, and 673 (18.5%) were gangrenous appendixes.

A total of 3117 (3117/4097, 76.1%) patients were monitored for complications at 7 days after the intervention. Major complications (Clavien-Dindo III–IV) [41] occurred in 199 patients (4.6%). A total of 287 patients (287/3117, 9.2%) developed complications at 7 days. Among these patients with complication, there were 60 with intra-abdominal abscesses (1.9%), 194 surgical site infections (6.2%), 6 paralytic ileus (0.2%), 6 seromas (0.2%), 9 other abdominal complications (0.3%), and 12 other medical complications (0.4%). A total of 2667 (2667/4097, 65.1%) patients were monitored for complication at 30 days after the intervention, and among them, 88 (88/2667, 3.3%) developed a complication. The complications occurred by 30 days were intra-abdominal abscesses (1.3%) in 35 cases, surgical site infections (1.9%) in 51 cases, paralytic ileus (0.1%) in 2 cases, other abdominal complications (0.2%) developed in 6 cases, and other medical complications (0.2%) in 5 cases. The overall mortality rate was 0.28%. The distribution of predictive variables of in-hospital mortality registered at univariate analysis is reported in Table 4. Independent variables associated with mortality according to the multinomial logistic regression are reported in Table 5.

**Table 4:** Distribution of predictive variables and mortality at univariate analysis

Variables	Patients *N* = 4282 (%)	Dead *N* = 12 (%)	Survivors *N* = 4270 (%)	RR (CI 95%)	*p* value
Age > 80 years	52 (1.2)	2 (16.7)	50 (1.2)	14.23 (3.90–51.96)	< 0.05
Immunosuppression	71 (1.7)	3 (25.0)	68 (1.6)	15.70 (5.73–43.01)	< 0.001
Severe cardiovascular disease	154 (3.6)	3 (25.0)	151 (3.5)	7.07 (2.62–19.07)	< 0.05
Charlson Comorbidity Score > 5	23 (0.5)	4 (33.3)	19 (0.4)	74.91 (29.93–187.48)	< 0.001
Previous episodes suspected app.	427 (10.0)	2 (16.7)	425 (10.0)	1.67 (0.47–5.95)	0.33
Previous antimicrobial therapy	327 (7.6)	4 (33.3)	323 (7.6)	4.40 (1.97–9.87)	< 0.05
WBC > 10,000/ml	3434 (80.2)	10 (83.3)	3424 (80.2)	1.04 (0.81–1.33)	1.00
CRP > 50 mg/l	876 (20.5)	5 (41.7)	871 (20.4)	2.04 (1.04–4.00)	0.08
WSES Grading System					
Grade 0	145 (3.4)	0	144 (3.4)	0	1.00
Stage 1	1896 (44.3)	3 (25.0)	1893 (44.3)	0.56 (0.21–1.50)	0.18^a^
Stage 2a	632 (14.8)	0	632 (14.8)	0	0.23
Stage 2b	129 (3.0)	1 (8.3)	128 (3.0)	2.78 (0.42–18.29)	0.31
Stage 3a	332 (7.8)	0	332 (7.8)	0	0.62
Stage 3b	181 (4.2)	0	181 (4.2)	0	1.00
Stage 3c	73 (1.7)	2 (16.7)	71 (1.7)	10.02 (2.77–36.27)	< 0.05
Stage 4	376 (8.8)	6 (50.0)	370 (8.7)	5.75 (3.24–10.22)	< 0.001
Not reported	517 (12.1)	0	517 (12.1)	NA	NA
Pathology					
Normal	144 (3.4)	0	144 (3.4)	0	1.00
Inflammation	1147 (26.8)	2 (16.7)	1145 (26.8)	0.62 (0.17–2.20)	0.53
Periappendicitis	236 (5.5)	1 (8.3)	235 (5.5)	1.51 (0.23–9.92)	0.49
Suppurative	1176 (27.5)	2 (16.7)	1174 (27.5)	0.61 (0.17–2.15)	0.53
Gangrenous	673 (15.7)	2 (16.7)	671 (15.7)	1.06 (0.30–3.76)	1.00
Perforation	256 (6.0)	4 (33.3)	252 (5.9)	5.65 (2.51–12.68)	< 0.05
Not reported	650 (15.2)	1 (8.3)	650 (15.2)	NA	NA

All *p* values calculated using two-sided Fisher’s exact test unless otherwise noted

*RR* risk ratio, *CI* confidence interval, *NA* not applicable

^a^Two-sided chi-square test

**Table 5:** Results of multinomial logistic regression for the analysis of variables associated with mortality

Variables	OR	95% CI	*p* value
Age > 80	4.54	0.59–35.20	0.15
Immunosuppression	1.17	0.11–12.63	0.90
Severe cardiovascular disease	1.51	0.25–9.08	0.65
Charlson Comorbidity Score > 5	52.45	5.95–462.03	< 0.05
Previous antimicrobial therapy	2.29	0.48–10.85	0.30
WSES Grading System			
Stage 3c	11.77	1.37–100.78	< 0.05
Stage 4	11.32	2.18–58.62	< 0.05
Pathology			
Perforation	1.43	0.28–7.24	0.66

*CI* confidence interval, *OR* odds ratio

## Discussion

AA is one of the most commonly occurring clinical challenges for emergency surgeons, because of its diagnostic work-up. The clinical presentation of AA may vary widely from mild symptoms, like moderate abdominal pain or fever, to most severe scenarios, such as diffuse peritonitis and sepsis [42]. The most frequent clinical symptom is right lower quadrant abdominal pain. If fever with chills is present, systemic involvement should be suspected. However, these symptoms are not specific for AA, since they can be present in other septic conditions, like infectious gastrointestinal disorders or genitourinary tract disorders in young female patients [41]. The median age of 29 years demonstrates the prevalence of this disease in young population. Our data showed that right lower quadrant pain and tenderness were the most frequently reported symptoms (91.2 and 69.6%), followed by vomiting, fever, and diffuse tenderness (42, 24.7, and 11.7%, respectively). Laboratory findings showed a high prevalence of white blood count (WBC) > 10,000 cells/ml (80.2%) and C-reactive protein (CRP) > 10 mg/L in 46.1% of cases. As reported in various studies, WBC and CRP are the most significant laboratory markers to be considered in case of AA. A WBC cut-off > 10,000/ml has a range of sensitivity between 65 and 85% and specificity between 32 and 82%, and CRP values > 10 mg/L have a range of sensitivity between 65 and 85% and specificity between 59 and 73% [43].

Imaging plays a cardinal role in the diagnosis of AA. Reliable imaging in patients with clinical suspicion of appendicitis results in reducing the rate of negative appendectomy by almost 15%. The most commonly used imaging techniques are ultrasonography (US), computed tomography (CT), and magnetic resonance imaging (MRI) [43]. In our study, about one third (28.8%) of patients did not undergo any radiological examination, whereas the majority (43.3%) underwent US and only 21.2% had a CT scan. The data demonstrates that often an accurate clinical examination supported by laboratory findings can help the surgeon to manage AA. However, in some cases, a radiological confirmation of the clinical suspicion is paramount, and when US is not sufficient for definitive diagnosis or there is no availability of US-specialized radiologists (i.e., during night-time in some hospitals), a CT scan would be the ideal option, with a sensitivity of 98.5% and a specificity of 98% [44, 45].

Different prognostic scores have been proposed for the clinical evaluation of AA. Alvarado and Appendicitis Inflammatory Responses (AIR; also called Andersson’s score) scores are the most commonly used and validated, being the result of a combination of clinical and biochemical variables with a significant predicting value [4]. Alvarado score has a sensitivity and specificity of 99 and 43% to rule out the diagnosis of AA when < 5 and a sensitivity of 82% and specificity of 81% if < 7. Andersson’s score has a sensitivity of 96% to rule out AA when < 4 and a specificity of 99% to diagnose appendicitis when > 8 [46]. In our study, Alvarado and Andersson’s scores were recorded in 90.1 and 87.6% of cases, respectively. The Alvarado Score was ≥5 in 3132 (89.8%) cases of AA confirmed by pathologic exam (RR = 1.11, 1.07–1.15 CI 95%, *p* < 0.001), while Andersson’s Score was ≥ 5 in 2736 (83.4%) cases of AA confirmed by pathologic exam (RR = 1.11, 1.07–1.14 CI 95%, *p* < 0.001).

The WSES grading score [38] was extensively used (87.9%), and about half of the patients were grade 1. In this case, the appendix is inflamed and this is probably the most common situation for an emergency surgeon. WSES grade 1 is also a specific condition where, if the appendix has hyperemia, edema, and fibrin exudates, a significant plasma exudation into the abdominal cavity may occur, with 10% risk of presence of gram-negative bacteria [47]. Therefore, grade 1 appendicitis can sometimes be at risk of developing post-operative peritonitis or abscesses and this risk should be considered, since it is the most common situation recorded in our study. Another important result is the low incidence of WSES grade 0 cases (3.8%), which in the daily practice represents the absence of pathological findings in the appendix. These cases represent the so called “normal-looking appendix” [46]. Such results correlate well with the pathology report of 4% of normal appendixes.

The vast majority of patients in our study underwent surgery (95.7%). More than half the cases were performed laparoscopically (51.7%), 42.2% had open appendectomy, and only 4.3% patients did not receive any surgical treatment. Despite there being several reports in the literature regarding non-operative management of uncomplicated AA [13, 48, 49], this global snapshot from our study demonstrates how operative management still forms the backbone of treatment by surgeons.

Both laparoscopic and open approach are safe and effective techniques for the treatment of suspected AA. Both techniques are associated with good clinical outcomes and few complications [50]. The benefits of laparoscopic approach include reduced incidence of surgical site infections, shorter postoperative stay, less pain, reduced incidence of incisional hernias, and faster postoperative recovery and return to everyday activities, along with better cosmesis [24, 51, 52]. However, the traditional open approach is still widely used, probably due to reduced cost, shorter operative and anesthetic times, the increased risk of intra-abdominal abscess associated with laparoscopic appendectomies and a reduced requirement of higher surgical skill levels [23, 24, 53–55].

Recently, WSES recommended the use of broad spectrum antibiotics in case of complicated AA for a minimum duration of 3–5 days of antibiotic treatment [46], and no postoperative antibiotics for uncomplicated appendicitis. In our study, 80.9% of patients received at least one antibiotic during hospitalization for a median duration of 4 days. The most commonly used antiobiotic was metronidazole (58.2%) followed by second- and third-generation cephalosporines (37.0 and 17.2%, respectively). Penicillin with beta-lactam inhibitors were used only in 14.4% cases. The overall number of antibiotics administered (*n* = 5775) was different from the number of patients receiving antibiotics (*n* = 3463) since 2128 patients received a combined antimicrobial therapy. This data correlates with the intraoperative findings of positive cultures collected in 275 patients (6.4% of the total population). *Escherichia coli* (aerobic gram-negative) was found in most of the cultures (57.8%), followed by anaerobic bacteria (*Bacteroides* spp. 38.2%) and *Enterococcus faecalis* (aerobic gram-positive) in 15.3%. The overall number of isolated microorganisms (*n* = 392) was different from the number of positive cultures (*n* = 275) since from 87 positive cultures, more than one microorganism was isolated. These multiple isolations correlated with the use of multiple antibiotics in about 70% of cases.

The reported incidence of postoperative complications in literature ranges from 3 to 28.7% [56, 57]. The most common complications quoted in the literature are small bowel obstruction, surgical site infection, stump leakage, abdominal abscess, and stump appendicitis [58, 59]. In our study, no stump leakage or stump appendicitis have been reported and although it is reported in the literature as occurring in patients with complicated appendicitis [60], there is no clear evidence of their incidence. Surgical site infection was the most common complication both at 7 and at 30 days after operation (6.2 and 1.9% incidence, respectively) and the reported incidence from the literature ranges between 1.2 and 12%. Small bowel obstruction is reported to have an incidence of around 2%, but in our study its occurrence was as low as 0.2% at 7 days and 0.1% at 30 days, much less than the rates reported in the literature. Abdominal abscesses are the second most frequent complication, with an incidence between 1.6 and 8% [59, 60]. Our study correlated with the incidence of abdominal abscesses reported in the literature with 1.9% at 7 days, but it was much lower at 30 days (1.3%). Complications were not recorded for 100% of the cases included in the study, and this represents a potential bias.

Histopathology showed 32.4% were suppurative appendixes, 31.6% inflammatory, and 18.5% gangrenous appendixes. These represent the majority of pathological diagnosis in case of AA, and they correlate well with preoperative diagnosis and scores registered in our study, but further analyses are necessary to better investigate the correlation between preoperative variables and intra−/postoperative findings. Unfortunately, the present study does not report any data on the incidence of appendiceal tumors, although this finding occurs in about 3% of all appendectomies in the literature [61, 62].

The median hospitalization of 3 days was less than the average length of hospital stay reported in literature [63]. The overall mortality rate following appendectomy in cases of complicated appendectomy can be up to 2.4% [2, 3], and the value of 0.28% is lower than the data reported in literature.

In the bivariate analysis, we have investigated the predictive factors of mortality. Significant variables were age > 80 years, immunosuppression, severe cardiovascular disease, Charlson Comorbidity Score > 5, previous episodes of suspected appendicitis, previous antimicrobial therapy, WSES stages 3c–4, and a pathologic report of perforation. At multivariate analysis, only Charlson Comorbidity Score > 5 (OR 52.45, *p* < 0.05) and WSES stages 3c (OR 11.77, *p* < 0.05) and 4 (OR 11.32, *p* < 0.05) were confirmed as independent variables that were predictors of mortality. Therefore, the presence of serious comorbidities is associated with significantly worse prognosis for such a benign disease, even in absence of complications. Surprisingly, perforation was not an independent risk factor associated to mortality; nonetheless, this should be interpreted with caution, due to the extremely low number of deaths.

## Conclusions

The results of the present study gives a snapshot of current worldwide trend in the diagnostic work-up and therapeutic management of AA. Ultrasound is used in about 40% cases and CT in one third. Alvarado, Andersson’s, and WSES grading scores are useful methods to classify the patients, and they predict and correlate with the surgical or pathological diagnosis. More than 90% of patients are treated with surgery, which, in more than 50% cases, is performed using a laparoscopic approach, with a low conversion rate. The hospital stay is usually short, with few complications at 7 and 30 days postoperatively. Even if it is low, the mortality rate seems to correlate with the presence of relevant comorbidities in cases of complicated appendicitis with abscess or peritonitis. Further analysis based on the present data are needed to study in detail the role of preoperative diagnostic work-up, the usefulness of prognostic scores, the potential value of appropriate antibiotic therapy, and the real advantages of a laparoscopic approach.
